# Full‐House Nephropathy Without Lupus Manifesting During Acute Kidney Allograft Rejection: A Case Report

**DOI:** 10.1002/ccr3.71762

**Published:** 2025-12-30

**Authors:** Tala Pourlak, Farahnoosh Farnood

**Affiliations:** ^1^ Department of Pathology Tabriz University of Medical Science Tabriz Iran; ^2^ Kidney Research Center Tabriz University of Medical Sciences Tabriz Iran

**Keywords:** acute allograft rejection, case report, full‐house immunofluorescence pattern, kidney transplantation

## Abstract

Kidney transplantation increases the survival rate of end‐stage renal disease patients; however, acute rejection and glomerulonephritis, such as the uncommon non‐lupus full‐house nephropathy (NLFHN), can lead to graft dysfunction. NLFHN exhibits a characteristic lupus immunofluorescence pattern in the absence of systemic lupus features, which makes both diagnosis and treatment more challenging for transplant recipients. A 34‐year‐old female with a background of ABO‐compatible living‐related kidney transplantation presented 12 years after the transplant with worsening hypertension, elevated serum creatinine (1.9 mg/dL), and subnephrotic range proteinuria. Biopsy of kidney revealed Non‐lupus full‐house nephropathy (NLFHN) accompanied by T‐cell‐mediated rejection. Immunofluorescence showed a “full‐house” pattern (IgG, IgA, IgM, C3, and C1q) in the absence of systemic lupus erythematosus (SLE) features (negative ANA, normal C3). Despite intensified immunosuppressive treatment, proteinuria persisted, highlighting challenges in managing overlapping immune‐mediated kidney pathologies. The simultaneous presentation of acute rejection and NLFHN indicates a complex interplay between alloimmune injury and immune complex deposition, contributing to graft damage. Management of this challenge, due to limited treatment options and variable responses, highlights the necessity for early detection and further research aimed at improving outcomes.

## Key Clinical Message

Patients with a history of kidney transplants who exhibit graft dysfunction alongside full‐house immunofluorescence may be diagnosed with non‐lupus full‐house nephropathy, a rare condition unrelated to lupus nephritis. Concurrent acute rejection complicates diagnosis and treatment. Correct biopsy analysis and personalized immunosuppression are essential for maintaining graft function and enhancing outcomes.

## 1 Introduction

Kidney transplantation provides higher quality of life and survival rate for end‐stage renal disease patients in comparison to those on dialysis [1]. Acute rejection (AR) is a major complication in kidney transplantation, adversely influencing the survival of the transplanted organ [2]. Acute kidney injury (AKI) in transplant patients necessitates a tailored diagnostic strategy, as it can arise from either typical causes or transplant‐related conditions [3]. The differential diagnosis consists of: 1‐Cellular or antibody‐mediated rejection 2‐Calcineurin inhibitor (CNI) toxicity 3‐Obstruction of urinary tract 4‐Vascular complications 5‐Recurrent or de novo glomerular disease 6‐BK polyomavirus nephropathy [4].

Recurrent and de novo glomerulonephritis following kidney transplantation are relatively common and represent important factors contributing to graft dysfunction and loss [5]. Among these, full‐house nephropathy (FHN) is characterized by the classical immunofluorescence pattern seen in lupus nephritis, including positive deposits of IgG, IgA, IgM, C3, and C1q. However, it lacks the systemic or serologic criteria required for diagnosing systemic lupus erythematosus (SLE), as established by the American College of Rheumatology (ACR) [6, 7].

Non‐lupus FHN creates diagnostic and therapeutic obstacles. It could represent either an initial or isolated renal manifestation of SLE or a separate pathological condition unrelated to SLE. Notably, less intense C3 deposition in non‐lupus membranous lupus nephritis (MLN) suggests a pathophysiology different from classical full‐house MLN.

The main differential diagnoses for non‐lupus FHN consist of lupus nephritis, atypical membranous nephropathy, IgA nephropathy, anti‐neutrophil cytoplasmic antibody (ANCA)‐associated glomerulonephritis, post‐infectious glomerulonephritis, and C1q nephropathy [8]. Additional conditions associated with full‐house nephropathy include liver disease, diabetes mellitus, infections such as HIV and hepatitis B or C, and C1q nephropathy [9]. While non‐lupus FHN has been observed in native kidneys, its occurrence alongside acute rejection in transplant kidneys is rare. This report aims to describe such a case and review relevant literature. Recognizing and understanding non‐lupus FHN within the transplant context is essential for precise diagnosis and management, ultimately enhancing graft outcomes.

## 2 Case Presentation

A 34‐year‐old woman with end‐stage renal disease secondary to hypertensive nephropathy underwent living donor renal transplantation 12 years ago. Since then, she has maintained stable graft function on a maintenance immunosuppressive regimen consisting of tacrolimus, mycophenolate mofetil, and low‐dose prednisone.

During routine follow‐up, laboratory tests revealed a gradual increase in serum creatinine, rising from her baseline of 1.7 to 2.4 mg/dL over the preceding 2 months. Concurrently, she has developed subnephrotic‐range proteinuria, quantified at approximately 2060 mg/24 h.

In clinical assessment, the patient denied fever, rash, arthralgia, hematuria, or recent infections. Physical examination was unremarkable, with no edema or clinical signs suggestive of systemic lupus erythematosus. Serological evaluation, including antinuclear antibody (ANA) and anti‐double‐stranded DNA (anti‐dsDNA) testing, was negative. Complement levels (C3 and C4) remained within normal range. Viral serology, including BK virus PCR, was also negative. Blood pressure was controlled at 130/80 mmHg. In the context of unexplained graft dysfunction, a percutaneous renal allograft biopsy was performed for histologic investigation.

## 3 Pathological Findings

Two cortical tissue fragments were examined, measuring 0.7 cm (preserved in saline) and 1.3 cm (fixed in formalin), respectively. Nineteen glomeruli were identified; globally scleroses were identified in 5 of them.

**FIGURE 1:**
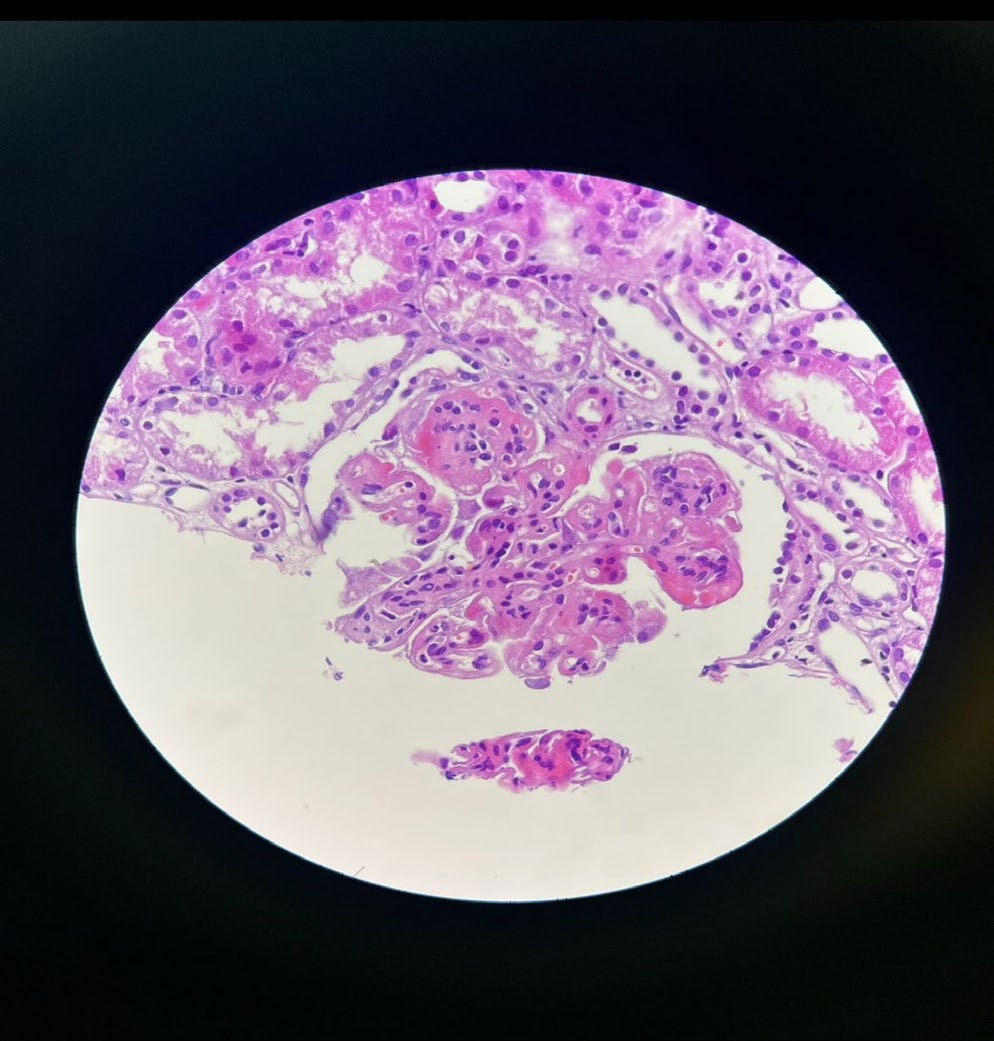
Glomerular Basement Membrane Alterations; In some glomeruli, duplication and splitting of the glomerular basement membrane, producing a characteristic “tram‐track” appearance, are observed. Variable mesangial expansion and areas of segmental capillary collapse are also evident.

**FIGURE 2:**
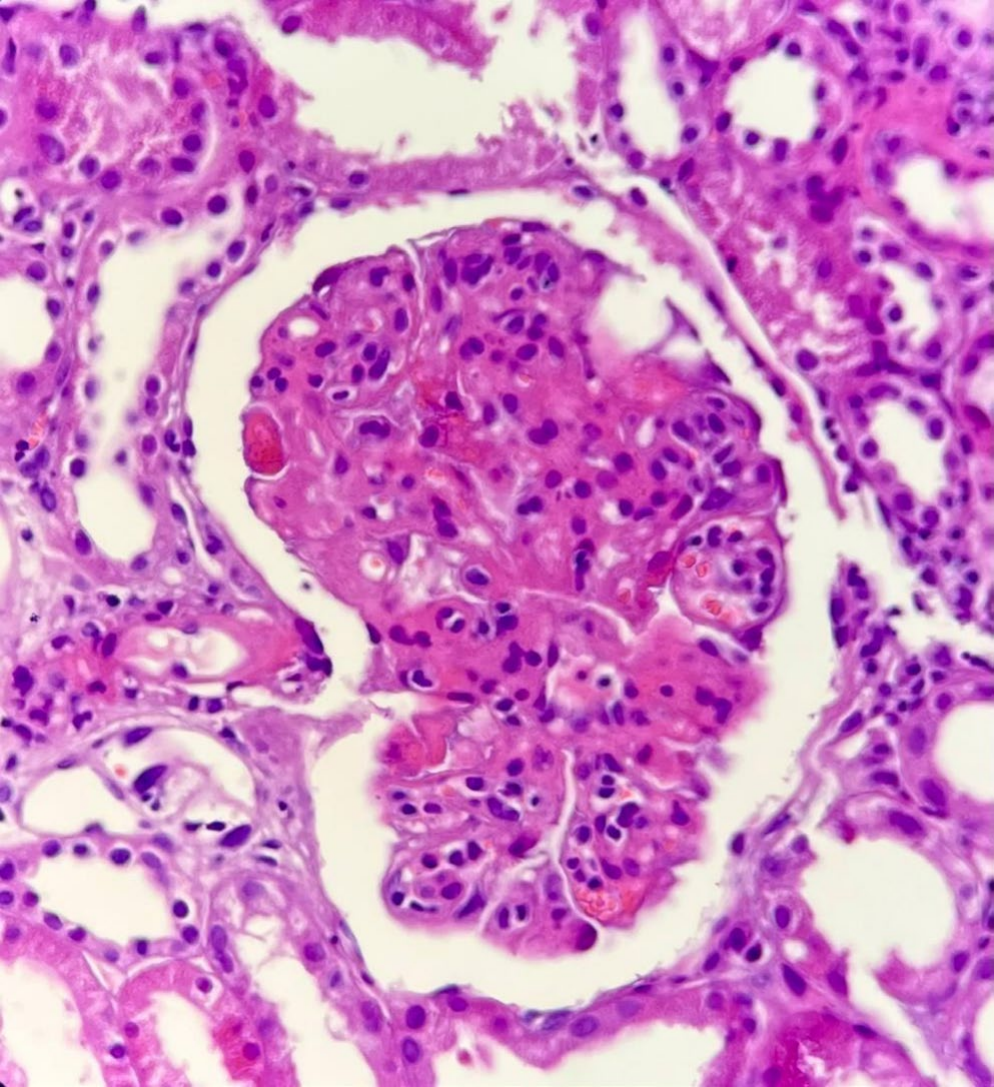
Glomerular Endothelial Injury and Capillary Occlusion; The glomerulus demonstrates prominent endothelial cell swelling with marked narrowing and focal occlusion of capillary lumina by eosinophilic thrombotic material.

In light microscopy, the glomeruli appeared enlarged with mesangial expansion accompanied by mild cellularity. Silver staining showed glomerular basement membrane (GBM) thickening and double contours formation in around 25%–50% of the glomeruli. Notably, there were no spikes or holes, and no evidence was detected for crescent formation, adhesions, fibrin deposits, endocapillary hypercellularity, necrosis, or capillary thrombi. The interstitium exhibited edema and focal lymphocytic infiltrates with tubulitis (3–5 lymphocytes per tubular cross‐section). Tubular injury with approximately 10% mild atrophy and proportional interstitial fibrosis was observed. Arterioles and arteries were largely unremarkable, without hyalinosis or vasculitis. No viral cytopathic changes were noted (Figures 1 and 2).

Immunofluorescence Microscopy: Demonstrated a full house pattern with global granular staining for IgG (3+), IgA (2+), IgM (2+), C1q (2+), C3 (2+), kappa (2+), and lambda (2+). Fibrinogen was negative.

Immunohistochemistry: C4d staining was negative in peritubular capillaries, and SV40 antigen was negative, excluding polyomavirus nephropathy.

## 4 Outcome

Non‐Lupus Full House Nephropathy (transplant glomerulopathy). Acute Cellular Rejection, TCMR (T‐cell mediated rejection) type I (Banff 2019 classification). Mild interstitial fibrosis and tubular atrophy (IFTA) involving ~10% of the sample. The patient was treated with a loading dose of oral methylprednisolone 500 mg for three consecutive days, followed by oral corticosteroids, according to the acute cellular rejection protocol. After stabilizing her general condition, she was discharged with a creatinine of 1.4 mg/dL.

The patient's serum creatinine improved from 1.9 to 1.4 mg/dL at discharge. She was continued on maintenance oral corticosteroids at 7.5 mg daily, along with baseline immunosuppression using tacrolimus and mycophenolate mofetil. Follow‐up includes regular monitoring of renal function, proteinuria, and immunologic markers to assess graft status and adjust immunosuppressive therapy as needed.

## 5 Discussion

Non‐lupus full‐house nephropathy (FHN) is a rare and exciting renal pathology defined by the presence of a full‐house immunofluorescence pattern‐deposition of IgG, IgA, IgM, C3, and C1q‐in the absence of systemic lupus erythematosus (SLE) clinical or serological criteria [6]. While full‐house staining is classically linked to lupus nephritis, multiple studies have reported similar immunopathological findings in other conditions, including infections, other autoimmune diseases, and idiopathic cases [10]. The presence of a full‐house immunofluorescence pattern in kidney transplants complicates diagnosis, as it usually suggests lupus nephritis but can occur without systemic lupus erythematosus (SLE) features. This leads to diagnostic uncertainty, making it difficult to distinguish between recurrent or new lupus nephritis, transplant glomerulopathy, and other immune complex‐mediated conditions [10, 11]. In the context of kidney transplantation, alloimmune injury can trigger inflammatory cascades that promote immune complex deposition. The coexistence of acute rejection and non‐lupus FHN in our patient raises the possibility that alloimmune activation during rejection episodes may precipitate or exacerbate glomerular immune complex deposition [12]. Conversely, immune complex‐mediated injury may amplify graft inflammation, creating a vicious cycle that threatens graft survival [12]. In our case, the absence of systemic lupus features (negative ANA, anti‐dsDNA antibodies, and normal complement levels) excluded classical SLE; these findings support a diagnosis of non‐lupus FHN rather than lupus nephritis recurrence.

Moreover, acute rejection features—tubulitis, interstitial inflammation, and C4d‐positive peritubular capillaries—were present, confirming concurrent alloimmune injury. Distinguishing immune complex‐mediated glomerulopathy from rejection‐related glomerular changes is essential but often requires a combination of clinical, serologic, and histologic data.

In pediatric patients, severe immune dysregulation in IPEX syndrome (Immune dysregulation, polyendocrinopathy, enteropathy, X‐linked syndrome), caused primarily by mutations in the FOXP3 gene located on the X chromosome, leads to defective regulatory T cell function and widespread autoimmunity. This immune dysregulation is involved in the pathomechanism of non‐lupus full‐house nephropathy, a rare cause of nephrotic syndrome in childhood [13].

In another article the development of non‐lupus full‐house nephropathy (FHN) during treatment of Crohn's disease with adalimumab highlights the importance of recognizing full‐house nephropathy beyond lupus, especially in patients with Crohn's disease [14].

In another study, the coexistence of heterozygous CFHR3‐CFHR1 deletion and full‐house nephropathy with an IgAN‐like pattern on immunofluorescence staining is reported. This indicates the diversity in the pathogenesis of non‐lupus FHN and warrants further investigation [15].

Non‐lupus FHN has variable outcomes in native kidneys, with some patients achieving remission and others progressing to chronic kidney disease. In transplant recipients, prognosis is further complicated by rejection episodes and the risks associated with intensified immunosuppression.

Current treatment options are lacking, with mixed results in the literature for varied immunosuppressive regimens. Rituximab, a selective anti‐CD20 B‐cell monoclonal antibody, has shown success in immune‐complex mediated glomerular diseases but has not been commonly used or studied in non‐lupus full‐house nephropathy [16]. A recent study described a case of non‐lupus full‐house nephropathy that was refractory to first‐line immunosuppressants, in which two rounds of rituximab treatment achieved a positive response [16, 17]. This case underscores the importance of recognizing non‐lupus FHN as a distinct entity in transplant nephrology. Early diagnosis may prevent misclassification as lupus nephritis and avoid unnecessary or inappropriate therapies. Further research is needed to elucidate the immunopathogenesis of non‐lupus FHN, identify biomarkers for diagnosis and prognosis, and develop targeted treatments.

## 6 Conclusion

This case underscores the importance of considering non‐lupus full‐house nephropathy in transplant recipients presenting with graft dysfunction and full‐house immunofluorescence, particularly when accompanied by acute rejection. Further studies are needed to elucidate pathogenesis and guide therapy. In summary, non‐lupus full‐house nephropathy coexisting with acute rejection represents a rare but significant cause of allograft dysfunction. Multidisciplinary evaluation integrating clinical, serological, and histopathological data is essential for accurate diagnosis. Tailored immunosuppressive regimens balancing control of rejection and immune complex‐mediated injury are critical to preserving graft function. Renal biopsy remains essential in establishing the diagnosis and guiding appropriate management.

## Author Contributions

**Tala Pourlak:** conceptualization, data curation, writing – original draft, writing – review and editing. **Farahnoosh Farnood:** methodology, project administration, writing – original draft, writing – review and editing.

## Funding

The authors have nothing to report.

## Ethics Statement

The study protocol was reviewed and approved by the ethics committee of the Tabriz University of Medical Science (IR.TBZMED.REC.1404.178).

## Consent

Written informed consent was secured from the patient.

## Conflicts of Interest

The authors declare no conflicts of interest.

## Data Availability

All data produced throughout this case report are openly available.
